# Toxicity Effects of Polystyrene Nanoplastics with Different Sizes on Freshwater Microalgae *Chlorella vulgaris*

**DOI:** 10.3390/molecules28093958

**Published:** 2023-05-08

**Authors:** Qingqing Xiang, Ying Zhou, Chengxia Tan

**Affiliations:** 1 College of Chemical Engineering, Zhejiang University of Technology, Hangzhou 310014, Chinatanchengxia@zjut.edu.cn (C.T.); 2 Environmental Microplastic Pollution Research Center, Zhejiang University of Technology, Hangzhou 310014, China

**Keywords:** polystyrene nanoplastics, *Chlorella vulgaris*, toxicity effects, aggregation

## Abstract

The ubiquitous nature of plastics, particularly nanoplastics, raises concern about their potential effects on primary producer microalgae. Currently, the impacts and potential mechanisms of nanoplastics on microalgae are not fully understood. In this study, the effects of two plain commercial polystyrene nanoplastics (PS-NPs) with different sizes (50 nm and 70 nm) on *C. vulgaris* were assessed in a concentration range of 0–50 mg/L during 72 h exposure periods. Results revealed that both PS-NPs have dose-dependent toxicity effects on *C. vulgaris,* as confirmed by the decrease of growth rates, chlorophyll a and esterase activities, and the increase of ROS, MDA, and membrane damage. The membrane damage was caused by the agglomeration of PS-NPs on microalgae and may be the key reason for the toxicity. Compared with 70 nm PS-NPs (72 h EC_50_ >50 mg/L), 50 nm PS-NPs posed greater adverse effects on algae, with an EC_50–72h_ of 19.89 mg/L. FTIR results also proved the stronger variation of macromolecules in the 50 nm PS-NPs treatment group. This phenomenon might be related to the properties of PS-NPs in exposure medium. The lower absolute zeta potential value of 50 nm PS-NPs induced the stronger interaction between PS-NPs and algae as compared to 70 nm PS-NPs, leading to severe membrane damage and the loss of esterase activity as well as settlement. These findings emphasized the importance of considering the impacts of commercial PS-NPs properties in toxicity evaluation.

## 1. Introduction

Plastic debris is an escalating environmental crisis and has been detected in nearly all aquatic ecosystems [1,2,3,4]. Reportedly, more than 300 million tons of plastic was produced annually in the world, and approximately 10% plastic was released into freshwater or the ocean [3,4,5]. Larger plastic debris can break down into microplastics(MPs) (1–5 mm) or nanoplastics (NPs) (1–1000 nm) via physical, chemical, and biological processes [1,6]. These small-sized plastics are abundant in aquatic environments [6,7]. Just on the surface of the Atlantic Ocean, the mass of three common MPs (polyethylene, polypropylene, and polystyrene) with the size of 32–651 µm reaches 11.6–21.1 million tons [7]. The abundance of MPs in remote areas such as Miri River (Borneo Island) can reach up to 2.1 mg/L or 14.3 particles/L [8]. Remarkably, MPs can continue decomposing into NPs [9,10], further increasing NPs pollution. The presence of NPs in aqueous environments has been demonstrated but not quantified owing to the limitation of analytical techniques [1]. It is estimated to be 10^14^ times higher than the presently measured abundance of MPs [11]. The vast accumulation of NPs causes growing concern about their potential effects to aquatic organisms due to their large surface area and the ability to penetrate cells [6,11].

Research on the aquatic toxicity of NPs has grown exponentially in recent years [6,12] with the majority using polystyrene (PS) as model NPs [13,14,15] due to their availability on the nanoscale [7]. Previous studies have shown that PS-NPs can pose a threat to various organisms such as fish, crustaceans, and zooplankton, affecting their growth, reproduction, and metabolism [16,17,18]. These studies are mainly focused on aquatic consumers (>70%), with relatively few investigations on primary producers [6]. Phytoplankton such as microalgae are the base of aquatic food webs and play a vital role in oxygen production, as well as the nitrogen and phosphorus biogeochemical cycle [19,20]. The minor disruptions of them may cause huge impacts to the entire ecosystem [18]. Therefore, the effect of NPs on microalgae merits more attention.

Moreover, the toxic impacts of NPs on algae are not well elucidated in existing studies. Some studies have documented that exposure to PS-NPs could inhibit algae growth and photosynthesis, even as induce oxidative damage [20,21,22]. These adverse effects generally increase with decreasing PS-NPs size [19]. However, the opposite results also exist. For example, Sendra et al. [23] found that particle size did not seem to substantially affect their toxicity; the smaller PS-NPs (50 nm) induced greater effects toward marine diatoms at 24 h, while the bigger ones (100 nm) were at 72 h due to the greater stability of them in exposure medium. Liu et al. (2019) reported that the inhibition effects of PS-NPs (100 nm and 500 nm) and PS-MPs (2 µm) on *Scenedesmus obliquus* growth were not size-dependent, but their effects on algae photosynthesis were enhanced with the increase of PS size [24]. In contrary to them, Sjollema et al. [25] indicated that PS-NPs with different size (50 and 500 nm) did not have obvious effects on algae photosynthesis even at the highest concentration of 250 mg/L. Given the contradictory findings and limited research, more investigations are urgently needed to better understand the potential effects and toxic mechanisms of NPs with different size on microalgae.

The aim of this study was to assess the potential effects of two plain commercial PS-NPs with different sizes (50 nm and 70 nm) on freshwater microalgae *Chlorella vulgaris* (*C. vulgaris*), in an attempt to elucidate the underlying toxicity mechanism of PS-NPs on microalgae. The effects of PS-NPs on *C. vulgaris* such as growth, photosynthesis, cell morphology, esterase activity, membrane damage, and oxidative stress were performed. Furthermore, the interaction between PS-NPs and microalgae was analyzed by FTIR. Considering the complexity of commercial PS-NPs, the behaviors of them in culture medium were also analyzed in order to better understand the toxicity effects of PS-NPs on algae. The results of this research are expected to provide useful information for assessing the impacts of nanoplastics on microalgae.

## 2. Results and Discussion

### 2.1. PS-NPs Characterization

The sizes, shapes, and chemical compositions of two PS-NPs were consistent with the information provided by supplier as verified by SEM (Figure S1) and FTIR (Figure S2). Both PS-NPs were spherical beads and without any surface coating. In order to better understand the behavior and biological impact of PS-NPs, more characterization about their physicochemical properties in exposure medium are needed prior to laboratory exposure, as suggested by previous studies [23,26]. Average hydrodynamic diameter and zeta potential are often used to elucidate the colloidal stability of nanoparticles in exposure medium [10,26,27]; these were also measured in the present study. As displayed in Table 1, the average hydrodynamic diameter of 50 nm PS-NPs in BG-11 was about eight times higher than their nominal size, with a value of 401.08 ± 15.62 nm. The average hydrodynamic diameter of 70 nm PS-NPs was slightly higher than the nominal size, with a value of 99.73 ± 0.49 nm. These data revealed that 50 nm PS-NPs aggregated more easily in BG-11 as compared to 70 nm PS-NPs, which was congruent with the observed PDI values (PDI > 0.2) that represented aggregation [26]. Regarding the charge, both PS-NPs exhibited negative charge in BG-11, with the values of −12.07 ± 0.65 mV and −35.28 ± 0.36 mV, respectively. Generally, particles with a zeta potential value from −30 mV to +30 mV are considered unstable in exposure medium [23,28]. The low absolute zeta potential of 50 nm PS-NPs further indicated the instability of them in BG-11.

**Figure 1:**
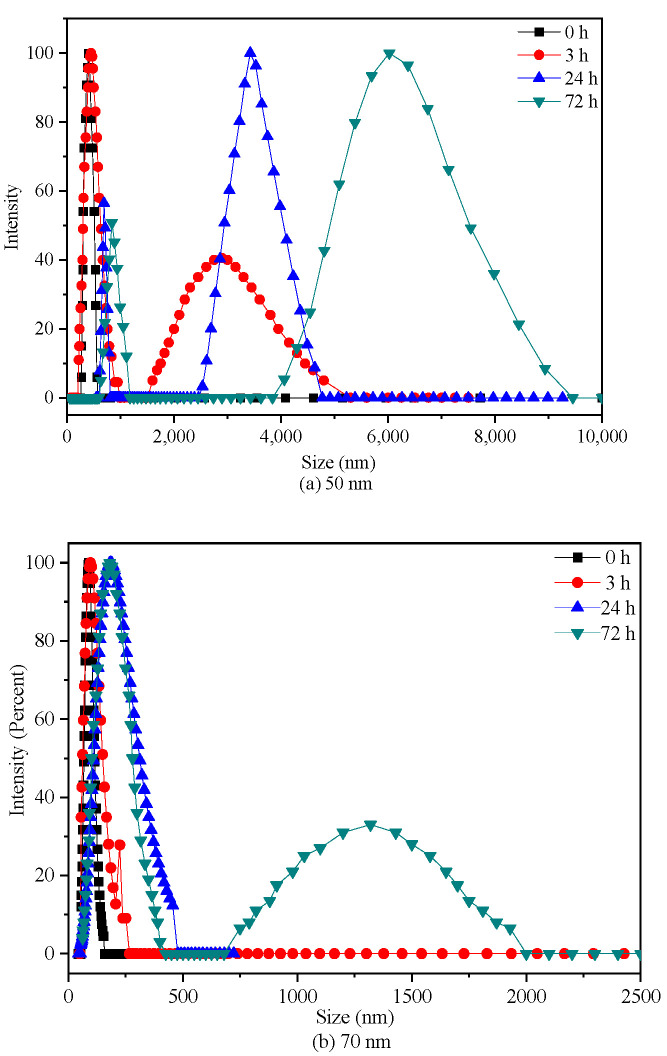
Intensity-based size distributions by DLS analysis of 50 nm (**a**) and 70 nm (**b**) PS-NPs (10 mg/L).

Considering the discrepancy in the stability of two PS-NPs in culture, their aggregation states were also analyzed by DLS after 3, 24, and 72 h exposure. As shown in Figure 1, an increase in size distributions of two PS-NPs were observed over times in BG-11, especially for 50 nm PS-NPs. Some particles in the 50 nm PS-NPs test group formed micron size (around 3 µm) during 3 h exposure period, while 70 nm PS-NPs was less variable and relatively stable. After 72 h exposure, 50 nm PS-NPs showed a maximum percentage at 6.5 µm, while 70 nm PS-NPs was mainly distributed around 250 nm, and few of them formed at micron scale (around 1.5 µm). Additionally, SEM analysis revealed that some of the 50 nm PS-NPs were deformed and formed some thin film fragments (Figure S1(A1)). Compared to these, 70 nm PS-NPs were spherical particles with minor change in diameter when they were exposed in BG-11 (Figure S1(B1)). These different behaviors of two PS-NPs might affect their toxicity effects toward algae, because several studies found that PS-NPs with less aggregation in the medium showed higher bioavailability [23,29]. Thus, this should be taken into consideration in toxicity evaluation.

### 2.2. Effects of PS-NPs on C. vulgaris Growth

**Figure 2:**
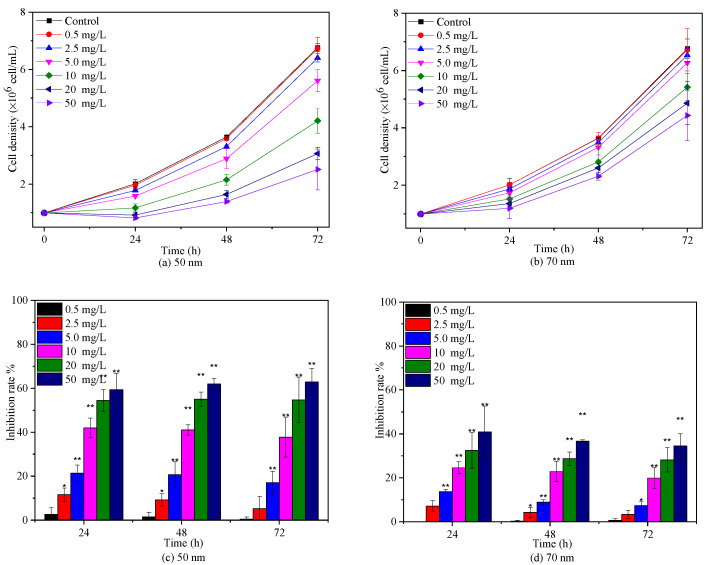
Effects of 50 nm (**a**,**c**) and 70 nm (**b**,**d**) PS-NPs on *C. vulgaris* growth during 72 h exposure durations. Significant differences between the control and treatments are marked with an asterisk (*: *p*< 0.05, **: *p* < 0.01).

The effects of 50 nm and 70 nm PS-NPs on *C. vulgaris* growth were studied at various concentrations between 0 and 50 mg/L, as presented in Figure 2. Both PS-NPs inhibited *C. vulgaris* growth during 72 h exposure periods, and the effect was enhanced with the increasing concentration, except for 0.5 mg/L. Similar dose–response negative effect of PS-NPs on freshwater microalgae growth in logarithmic phase was also observed by Mao et al. [19] and Liu et al. [24] at concentrations from 10 to 100 mg/L. With regard to two PS-NPs, *C. vulgaris* seemed to be more sensitive to 50 nm PS-NPs, with a 72 h EC_50_ for 19.89 mg/L (Table S1). The average cell density of *C. vulgaris* was significantly (*p* < 0.05) reduced by 17.08%, 37.75%, 54.69%, and 62.88% with respect to the control after exposure in 5, 10, 20, and 50 mg/L 50 nm PS-NPs for 72 h, respectively, while another PS-NPs-treated algae obviously (*p* < 0.05) declined by 7.33%, 19.89%, 28.14%, and 34.48% under the concentration of 5, 10, 20, and 50 mg/L, respectively. Some studies reported that the effect of PS-NPs on algae growth was increased with the decreased size [25]. Here, it didn’t seem to be a plausible explanation for the difference between two PS-NPs, because the average hydrodynamic diameter of 50 nm PS-NPs was higher than 70 nm PS-NPs in BG-11 (Table 1). Generally, the strong aggregation pattern of PS-NPs in the medium could reduce their bioavailability and toxicity [10]; however, this trend did not occur in the 50 nm PS-NPs treatment groups. This probably meant that the other toxic mechanism coexisted. Analogously, Sendra et al. [23] found that unstable 50 nm PS-NPs, which aggregated readily in the exposure medium, showed higher IR (67.1% ± 13.0%) on *Phaeodactylum tricornutum* growth than relatively stable 100 nm PS-NPs (54.1% ± 12.6%) at 10 mg/L after 72 h exposure. Likewise, a recent study found that 65 nm PS-NPs with larger aggregation size showed greater dose-dependent adverse effects on *Karenia mikimotoi* growth in comparison to 100 nm PS-NPs with less aggregation [30]. However, the reasons for this phenomenon are unclear and need to be further explored.

### 2.3. Effects of PS-NPs on Algae Photosynthesis

**Figure 3:**
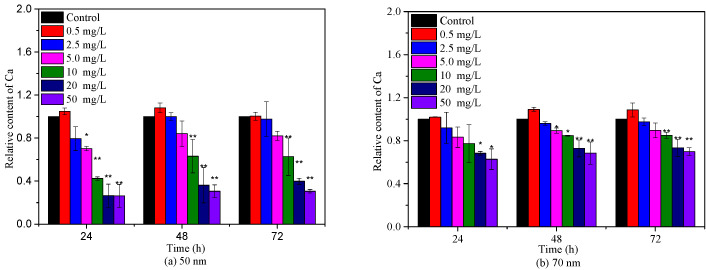
Effects of 50 nm (**a**) and 70 nm (**b**) PS-NPs on chlorophyll a during 72 h exposure durations. Significant differences between the control and treatments are marked with an asterisk (*: *p* < 0.05, **: *p* < 0.01).

**Figure 4:**
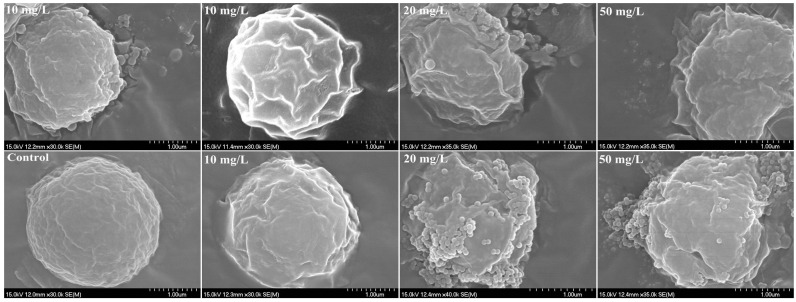
SEM images of *C. vulgaris* after 72 h exposure to 50 nm (**above**) and 70 nm (**below**) PS-NPs.

Algae growth is closely related to the chlorophyll content because chlorophyll is responsible for light absorption and energy transformation [31]. To further assess the toxicities of 50 nm and 70 nm PS-NPs to *C. vulgaris*, *Chl-a* was measured during 72 h exposure periods. As depicted in Figure 3, the relative contents of *Chl-a* showed a descending trend with the increasing concentration of 50 nm and 70 nm PS-NPs, except for 0.5 mg/L. Particularly, 50 nm PS-NPs exhibited the greater effect on *Chl-a*, with an EC_50–72 h_ value of 17.52 (13.31–24.72) mg/L (Table S1). However, the effect of another one was gradually diminished with time, with a maximum IR of 37.24% (EC_50–72 h_ >50 mg/L). These findings were consistent with the above-observed algae growth inhibition results, suggesting that PS-NPs could affect algae growth by perturbing algae photosynthesis, since the decline of *Chl-a* could hinder the metabolism and energy transfer of photochemical reactions [12]. In agreement with our findings, Hazeem et al. [27] and Wang et al. [32] reported that PS-NPs could cause a reduction in chlorophyll a concentration during the exponential growth phase, showing a direct effect on algae growth and photosynthesis. Similar negative effects of PS-NPs (50 nm and 100 nm) on chlorophyll a were observed by Sendra et al. [23], but 100 nm PS-NPs with less aggregation showed higher effect on *Chl-a* (EC_50–72 h_ = 11.5 mg/L) than unstable 50 nm PS-NPs (EC_50–72 h_ = 44.9 mg/L), which was contrary to our discovery. Different findings might be related to PS-NPs size or algae species. In this study, although 50 nm PS-NPs agglomerated more easily in the culture, these large aggregates or monodisperse 50 nm PS-NPs still could adhere on algae surfaces (Figure 4), which might affect algae photosynthesis by blocking the light transport [21]. A recent study found that the unstable PS-MPs (1 μm) was more greatly to affect algae photosynthesis than PS-NPs (100 nm) with less aggregation, which could support the observed result in this study [28].

### 2.4. Effects of PS-NPs on Intracellular Oxidative Stress and Esterase Activities

**Figure 5:**
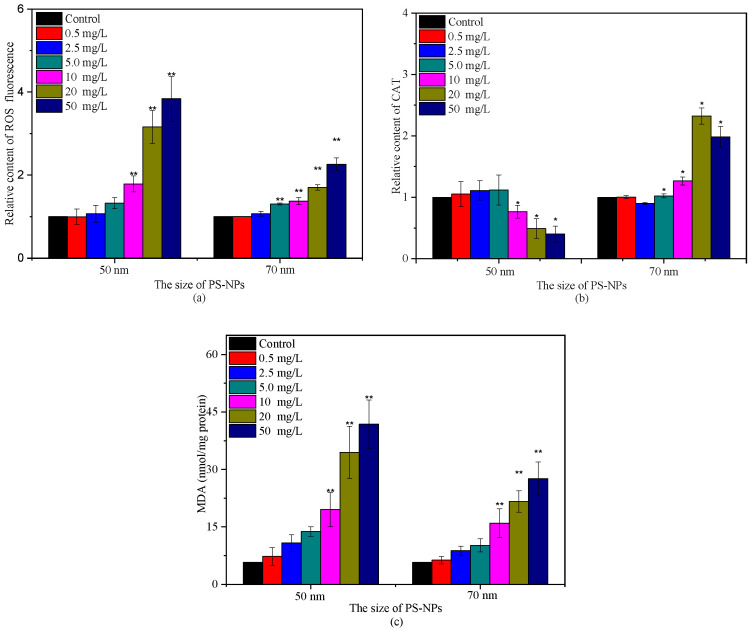
Variations of ROS relative content (**a**), CAT relative content (**b**), and MDA content (**c**) after treating with different concentrations of PS-NPs (* and ** denote significant (*p* < 0.05) and very significant (*p* < 0.01) differences from the control, respectively).

Algae as photosynthetic organisms can produce ROS during different metabolic pathways in mitochondria, chloroplasts, and peroxisomes under environmental stress [21,27]. The interference of PS-NPs on photosynthesis has been reported to enlarge intracellular ROS accumulation [21,28]. A similar phenomenon was observed in this study. The ROS and MDA levels of *C. vulgaris* showed positive responses with PS-NPs concentrations after 72 h incubation in two PS-NPs, especially in 50 nm PS-NPs treatment groups (Figure 5). The increased ROS and MDA contents indicated that PS-NPs could induce oxidative damage to *C. vulgaris.* Although antioxidant enzymes could participate in antioxidant protection processes [5,22], their activities could be reduced or even inhibited when ROS overproduction exceeded the inherent defense capacity of algal cells [12]. It can be seen from Figure 5b that antioxidant enzyme CAT activity was enhanced first then decreased with the increase of PS-NPs concentrations. Noticeably, *C. vulgaris* might have different tolerance for two PS-NPs. The CAT activity began to decline at 10 mg/L in 50 nm PS-NPs treatment groups, as compared to the control, while it was increased at high concentrations (≥10 mg/L) of 70 nm PS-NPs-treated groups, and a small decrease was observed at 50 mg/L relative to 20 mg/L. This discrepancy was probably associated with the higher ROS and MDA content that was induced by 50 nm PS-NPs as compared with 70 nm PS-NPs, since excessive ROS and MDA levels could destroy the intrinsic antioxidant defenses of cells and promote membrane lipid peroxidation [22,26]. These discoveries showed that 50 nm PS-NPs caused the stronger oxidative damage on *C. vulgaris* with respect to 70 nm PS-NPs.

**Figure 6:**
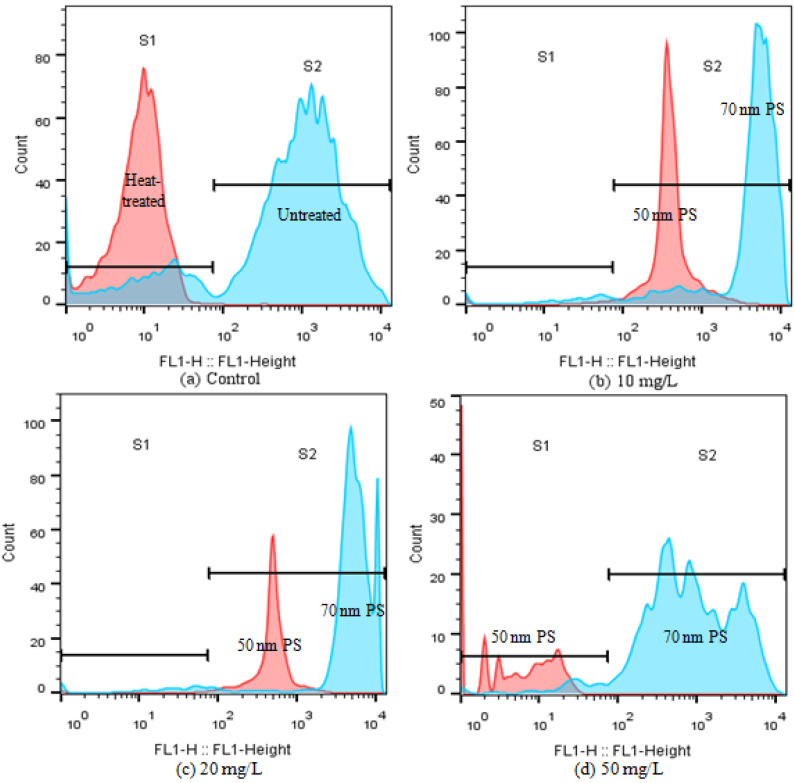
Alternation of esterase activity(FL1 fluorescence) of *C. vulgaris* after 72 h exposure to PS-NPs by flow cytometry: (**a**) control, (**b**) 10 mg/L, (**c**) 20 mg/L, (**d**) 50 mg/L.

Given the obvious difference of the two PS-NPs on CAT activity at high concentrations (≥10 mg/L), esterase activities were also analyzed in the present study, which is a critical biochemical parameter to assess microalgae metabolic activity [26,33]. FDA is a nonfluorescent, lipophilic molecule that can diffuse freely across the plasma membrane and produce a brightly fluorescent constituent called fluorescein (FITC) in viable cells [33]. Consequently, the intensity change of FITC fluorescence can reflect esterase activity. As shown in Figure 6, unheated control cells (negative control) were mainly distributed in S2, and their FITC fluorescence was 100-fold higher than that of heat-treated algal cells (positive control). After treating with the high concentrations of PS-NPs (≥10 mg/L), FITC fluorescence showed a dose-dependent reduction in 50 nm PS-NPs treatment groups, as compared with the negative control, while the FITC fluorescence of 70 nm PS-NPs was increased first then decreased. This phenomenon was similar to that observed in CAT. The rising of CAT activity and esterase activity reflected an activation and upregulation of detoxification processes as a stress response to pollutants [10,33], which meant that *C. vulgaris* could scavenge excess ROS that were induced by 70 nm PS-NPs at the high concentrations of 10 and 20 mg/L, while the esterase activity was inhibited at 50 mg/L. However, the CAT activity and esterase activity of *C. vulgaris* were inhibited at the high concentrations of 50 nm PS-NPs (≥10 mg/L), compromising the metabolic activity. Overall, the high intracellular ROS accumulation and the loss of esterase activity might be the cause for the greater effects of 50 nm PS-NPs on algae growth and photosynthesis.

### 2.5. Interactions between PS-NPs and C. vulgaris: The Variations of Algae Surface Macromolecular Composition and Cellular Structure

**Figure 7:**
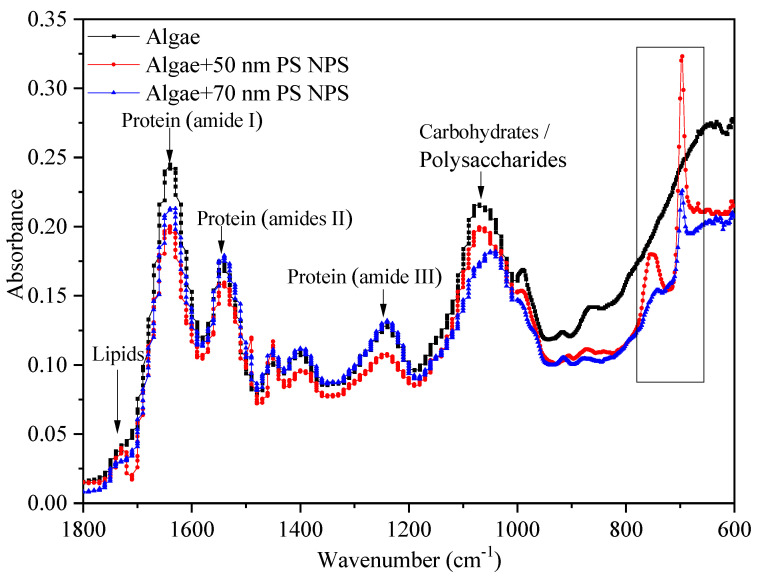
FTIR spectrums of *C. vulgaris* after treating with 20 mg/L PS-NPs (black line: control, red line: 50 nm PS-NPs, blue line: 70 nm PS-NPs).

Although both PS-NPs showed negative charges in exposure medium, they could adhere to microalgae surfaces by physical adsorption and/or weak chemical bonds, as previously reported [26,34]. This heteroaggregation may cause more interaction between particles and algae [19]. The surface of *C. vulgaris* was the first barrier for the interference of PS-NPs, and is mainly composed of proteins, lipids, and carbohydrates/polysaccharides [10,22,35]. They have specific characteristic bands in the FTIR spectrum, such as proteins (1650 cm^−1^, amide I), carbohydrates (1045 cm^−1^, C–O), and lipids (1742 cm^−1^, C=O) [36].These biomolecules play important roles in the stability of cell surface structure and function. The variations of them are vital signs in evaluating NPs toxicity [36]. Thus, the interactions between PS-NPs and algal cells were investigated by FTIR. As shown in Figure 7, the FTIR spectra of algal cells were altered in comparison to the control after treating with PS-NPs. Two additional absorption peaks (650–800 cm^−1^) appeared on algae surfaces under PS-NPs treatment, which were the monosubstituted benzene absorptions of PS-NPs (695, 750 cm^−1^) [27], revealing the attachment of PS-NPs on algae surfaces. Notably, the intensities of benzene-related peaks in the 50 nm PS-NPs treatment group were stronger than 70 nm PS-NPs. This might be due to the high negative charge value of 70 nm PS-NPs affecting the physical adsorption of PS-NPs on microalgae [26]. In addition, the lipid-related absorption peak (C=O, 1742 cm^−1^) emerged more clearly in the 50 nm PS-NPs treatment group than others, corresponding with the above-observed MDA result (Figure 5c). Moreover, the intensities of protein-related peaks (amide I 1650 cm^−1^, amide II 1540 cm^−1^,and amide III 1240 cm^−1^) and polysaccharide/carbohydrate-related peaks (1200–900 cm^−1^) were declined in the 50 nm PS-NPs treatment group with respect to the control. These reductions were connected with the cell membrane damage induced by NPs [15]. By contrast, the changes of macromolecules were slightly different in the 70 nm PS-NPs treatment group. The intensities of protein amide I peak (1650 cm^−1^) and polysaccharide-related peak (1045 cm^−1^) were decreased, but the protein amide II peak (1540 cm^−1^) and amide III peak (1240 cm^−1^) did not have apparent change, and a blue shift also appeared in the polysaccharide-related peak. These variations indicated that PS-NPs could interfere with algal surface biomacromolecules and thus affect cell surface structure.

**Figure 8:**
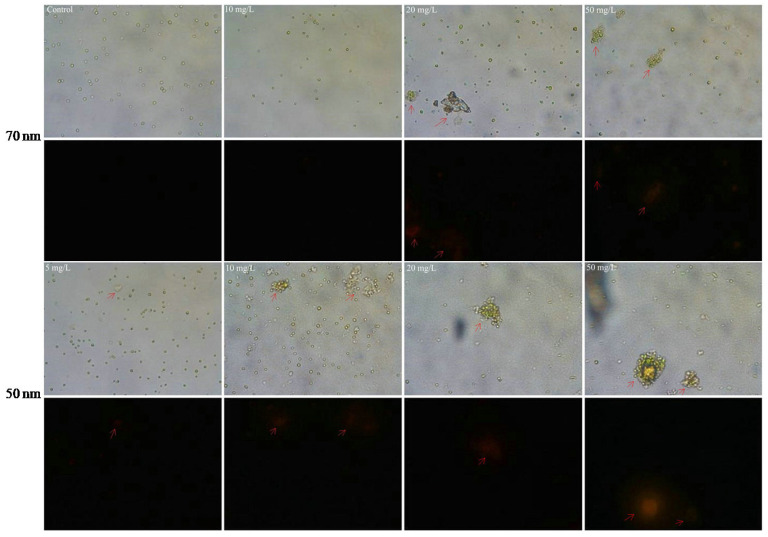
Microscope images of *C. vulgaris* after 72 h exposure to 50 nm (**lower**) and 70 nm (**upper**) PS-NPs (40× magnification). The fluorescent images (black part) were obtained under the excitation mode of green light source. Arrows show the aggregation of *C. vulgaris* and appear orange fluorescent.

In addition, the interactions between PS-NPs and algal cells were visualized by optical microscopes. SEM figures revealed that the formation of heteroaggregation between PS-NPs and algae could lead directly to physical damage. Compared with the smooth spherical algal cell (blank control), the irregular morphology of algae with some wrinkles was more pronounced with the increased adsorption of PS-NPs on algae surfaces (Figure 4). Membrane damage may be a possible consequence for the deformation of algae morphology [22]. To further verify this, membrane damages were analyzed by the AO-EB double-staining method. AO could pass through the normal cell membrane, while EB only could pass through the damaged cell membrane and produce orange fluorescence [37,38,39]. As presented in Figure 8, some algae cells were agglomerated and emitted bright orange–red fluorescence after treating with PS-NPs 72 h, which were more obvious with the increase concentration of PS-NPs, whereas the control and other well-dispersed algal cells did not emit orange–red fluorescence. Similar cellular aggregation induced by PS-NPs was also observed by Khoshnamvand et al. [40]. This phenomenon meant that PS-NPs might act as intermediaries for connecting algal cells, and thus forming aggregates, which was a dominant factor for the membrane damage. Particularly in the 50 nm PS-NPs treatment groups, algal cells were more likely to aggregate. At low concentration (≤10 mg/L), some aggregates were formed, while at the high concentration (≥20 mg/L), algal cells were mostly aggregated. By contrast, algal cells were less aggregated in 70 nm PS-NPs treatments, and partial aggregates were just observed at high concentrations (≥20 mg/L). This difference may be associated with their negative charge values, as the high negative charge values of 70 nm PS-NPs more readily led to the electrostatic repulsion between PS-NPs and algae [34]. These findings were consistent with FTIR results showing that 50 nm PS-NPs were more likely to interact with algal cells, resulting in the stronger adsorption on algal cell surfaces. The interaction of them could cause severe damage to cell membranes. This may be a reason for the higher toxicity of them on *C. vulgaris*.

As mentioned above, the formation of aggregation between PS-NPs and algae might be the main reason for the toxicity effects of PS-NPs on algae. It not only could lead to directly physical damage, but could also be associated with the decrease of algal photosynthesis and esterase activity as well as the increase of ROS [12,21]. Meanwhile, it could enhance the burden of algal cells and cause them to sink, thus affecting their growth [41]. A similar phenomenon was observed in this study, as shown in Figure S3. The settlement of algae was obviously accelerated when algae and PS-NPs coexisted in comparison to the control, particularly in the 50 nm PS-NPs treatment group.

## 3. Materials and Methods

### 3.1. PS-NPs Characterization and Stability Testing

Two monodispersed plain PS-NPs (50 nm and 70 nm) were purchased from BaseLine ChromTech Research Centre (Tianjin, China)as 2.5% *w/v* suspension dispersed in deionized water(10 mL). Both of them were stored in the refrigerator at 4 °C until use. Prior to use, they were ultrasonicated for at least 10 min. Particle shape, size, and surface chemical composition were verified by scanning electron microscopy (SEM) and Fourier-transform infrared spectroscopy (FTIR), respectively (Figures S1 and S2).

Hydrodynamic diameter, polydispersity index (PDI), and zeta potential analyses of PS-NPs were also performed in algae culture medium (BG-11) by a nano zeta potential and submicron particle size analyzer (Beckman Coulter Delsa™ Nano C, Beckmancoulter, CA, USA). Both PS-NPs were diluted to 10 mg/L, and then ultrasonicated for 10 min. Samples were immediately analyzed three times with the automatic mode at 25 °C. The hydrodynamic diameter distributions of PS-NPs in BG-11 were further measured by DLS at 3, 24, and 72 h to evaluate the stability of PS-NPs in BG-11. After 72 h exposure, the shape and morphology of PS-NPs were also analyzed by SEM.

### 3.2. Algal Culture and Growth Inhibition Test

*C. vulgaris* was purchased from the Institute of Hydrobiology, Chinese Academy of Sciences, and maintained in BG-11 medium. The algae were cultured at 24 ± 1 °C in an incubator under irradiance 81 µmol·m^−2^·s^−1^ with a 14:10 (light:dark) photoperiod. Exponential growth phase algal cells were cultured in 150 mL culture medium in the presence or absence of PS-NPs with series of dilutions (0, 0.5, 2.5, 5, 10, 20, and 50 mg/L) in 250 mL Erlenmeyer flasks for 72 h. The initial cell density of algae was 1 × 10^6^ cells/mL. Three replicates were performed for each concentration. To ensure optimum growth, cultures were shaken five times per day during incubation. Algal cell density was monitored at 685 nm by UV spectrophotometer (UNICO 2802 S, Franksville, Racine County, WI, USA) as well as counted with hemocytometer under optical microscope (Nikon, Tokyo, Japan) per 24 h. The regression equation for the relationship between cell density (y × 1.0 × 10^6^ cells/mL) and absorption at 685 nm (x) was calculated as y = 31.31x + 0.12 (*p* < 0.01, R^2^ = 0.99).

The algal growth inhibition ratio (IR) was calculated using the following equation:

(1) $$IR_{i}(\%)=(C_{ci}-C_{ti})/C_{ci}\times 100\%$$

where IR_i_ is the growth inhibition rate at time I, C_ci_ is the cell density of the control at time I, and C_ti_ is the cell density of the treated group at time i.

### 3.3. Quantification of Chlorophyll a

*C. vulgaris* cells were harvested by centrifugation (8000 rpm, 10 min) per 24 h. To avoid the possible interference of PS-NPs to UV, algae samples containing PS-NPs were washed with phosphate buffer (0.01 M, pH = 7.4) before chlorophyll extraction. Chlorophyll a (*Chl-a*) was then extracted with ethanol overnight [42,43] and analyzed by UV at 663 and 645 nm. The content of chlorophyll a (mg/L) was calculated by the formula C_a_ = (12.7 × OD_663_ − 2.69 × OD_645_) × V_1_ ÷ V_2_, where V_1_ (mL) and V_2_ (mL) were the volume of pigment extraction and microalgae solution, respectively. The data are expressed as relative content of the control.

### 3.4. Oxidative Stress Analysis

Algal cells were collected by centrifugation (8000 rpm, 10 min), and then resuspended in a phosphate buffer(0.01 M, pH = 7.4). To avoid potential interference of PS-NPs, the algal cells were properly washed with phosphate buffer and then were homogenized by sonication in an ice bath for 5 min. The homogenate was centrifuged and the supernatant was collected for further analysis. The proteins were detected by Coomassie bright blue G-250 method [44]. ROS was measured by conversion of nonfluorescent 2′7′-dichlorofluorescin diacetate (DCFDA) to the higher fluorescent compound dichlorofluorescein (DCF) as described by Wang and Joseph [45]. Fluorescence intensities were measured by SpectraMax M5 Microplate Reader (Molecular Devices, USA) under the condition of 488 nm excitation and 530 nm emission wavelengths. Malondialdehyde (MDA), a molecular indicator of lipid peroxidation [22], was evaluated using a microscale MDA assay kit (Nanjing jiancheng Bioengineering Institute, Nanjing, China) according to the manufacturer’s protocol. Catalase (CAT) activity was also measured using a CAT assay kit (Nanjing jiancheng Bioengineering Institute, Nanjing, China). All biomarker concentrations were normalized to their protein content, respectively. Each experiment was conducted with three replicates.

### 3.5. Esterase Activity Analysis

Esterase activity of *C. vulgaris* was assessed using the fluorescein diacetate (FDA) method and measured by flow cytometry (FACScalibur; BD Biosciences, Franklin Lakes, NJ, USA). The staining protocols for *C. vulgaris* were performed according to previous studies [33,46]. After 72 h exposure, algal cells were collected by centrifugation (8000 rpm, 10 min), then washed with 0.01 M PBS. The collected algae were stained with FDA at final concentrations of 25 µM, and incubated for 20 min in the dark. Esterase activity was determined by FL1 channel (500–560 nm bandpass filter, excitation at 488 nm blue laser, 15 mV, argon ion laser) after staining with FDA. Data were analyzed using Flowjo_V10 software.

### 3.6. Characterization of the Interactions between PS-NPs and Algae by FTIR

FTIR analysis was conducted to assess the change of biomacromolecules on algae surfaces after interacting with PS-NPs, which can be used to evaluate the degree of cell damage [27]. A total of 20 mg/L PS-NPs-treated or untreated algae were prepared by an identical procedure for FTIR analysis [27,47]. In brief, algal cells from 10 mL suspension were harvested by centrifugation for 10 min at 8000 rpm after 72 h exposure. Then, the cells were washed with PBS twice, and naturally dried for one day at 25 °C. The dried cells were analyzed by ATR-FTIR (Nicolet 6700, Thermo, Waltham, MA, USA).A spectral range from 4000 to 500 cm^−1^ was collected with an accumulation of 10 scans and resolution of 4 cm^−1^. To eliminate the effect of water-related O–H chemical bond (3500–3200 cm^−1^), the spectral area chosen for following analyses was restricted to 1800–600 cm^−1^ [48].

### 3.7. Analysis of Cell Morphology

The morphology of *C. vulgaris* was examined by SEM (Hitachi S-4700 (II), Japan) upon PS-NPs treatment. Algal cells were collected by centrifugation (8000 rpm, 10 min) after 72 h exposure, and then washed with PBS (0.01 M, pH 7.4) twice. The collected algal cells were fixed with 2.5% glutaraldehyde overnight at 4 °C, and then washed with PBS again. After that, the samples were dehydrated in a series of ethanol solutions (30%, 50%, 70%, 80%, 90%, 95%, and 100%) [22]. Afterwards, the cells were dispersed in a conductive adhesive by 10 µL pipette (Rainin, Onkland, CA, USA) and naturally dried for 2 h. The dried samples were sputtered with gold layer and analyzed by SEM.

### 3.8. Membrane Damage Analysis by Fluorescent Microscopy

To evaluate the cell damage induced by PS-NPs, algal cells were stained with DNA-binding dyes acridine orange (AO) and ethidium bromide (EB) double-staining method according to the method described by previous studies [37,38,39], After 72 h exposure, cell suspensions were mixed with AO/EB solutions (100 mg/L AO in PBS, 100 mg/L EB in PBS) for 30 min at final concentrations of 2 mg/L AO and 5 mg/L EB. The algal cells were immediately dropped into a glass slide and analyzed under green excitation light by fluorescence inverted microscope (Nikon, Tokyo, Japan).

### 3.9. Statistical Analysis

Data were expressed as the mean ± standard deviation. EC_50_ values (the effective concentration of PS-NPs that causes an inhibition of 50% microalgae growth) were computed using probit analysis. The differences between control and test groups were analyzed by one-way ANOVA and followed by LSD post hoc test. Significant differences were considered at *p*< 0.05. All the above analyses were performed using IBM SPSS Statistics 20.0.

## 4. Conclusions

This study investigated the effects of PS-NPs with different sizes (50 nm and 70 nm) on *C. vulgaris.* The results showed that both PS-NPs have acute toxicity on *C. vulgaris*. The interaction between PS-NPs and algae (such as adsorption and aggregation) was probably the main reason for the toxic effect of PS-NPs on algae. Comparing with 70 nm PS-NPs, 50 nm PS-NPs posed the greater deleterious effects on microalgae in terms of algae growth, photosynthesis, esterase activity, and cell morphology. FTIR further proved the interaction of algal surface groups with 50 nm PS-NPs, and the adsorption of NPs on algae surface. In addition, fluorescence microscopy presented the evidence that 50 nm PS-NPs more readily promote the formation of algal aggregates, causing severe damage to cell membranes. The discrepancy in toxicity between the two PS-NPs may be related to their properties in the exposure medium. The lower absolute zeta potential value of 50 nm PS-NPs might lead to more interaction between PS-NPs and algae as compared to 70 nm PS-NPs, thus resulting in the severe damage of cell membranes and the loss of esterase activity as well as settlement. These findings emphasize the importance of considering the impacts of commercial PS-NPs properties in toxicity evaluation.

## Figures and Tables

**Table 1:** Characterization of PS-NPs in BG-11.Values are shown as mean ± standard deviation of the three measurements.

Material	Diameters (nm)	PDI	ζ Potential (mV)
50 nm PS	401.08 ± 15.62	0.27 ± 0.01	−12.07 ± 0.65
70 nm PS	99.73 ± 0.49	0.15 ± 0.12	−35.28 ± 0.36

## Data Availability

Data are available from the corresponding author upon request.

## Supplementary Materials

The following supporting information can be downloaded at: https://www.mdpi.com/article/10.3390/molecules28093958/s1, Figure S1: The SEM images of 50 nm PS-NPs (left) and 70 nm PS-NPs (right) (above: raw materials; below: PS-NPs after 72 h exposure in BG-11, red arrows represent the deformation of PS-NPs). Figure S2: FTIR characterization of primary 50 nm and 70 nm PS-NPs. Figure S3: Effect of PS-NPs on sedimentation for algal cell (20 mg/L PS-NPs). Table S1: The EC50 values for algae growth and chlorophyll a after exposing to 50 and 70 nm PS-NPs (0–50 mg/L) for 72 h.

## Sample Availability

Samples are available from the corresponding author upon request.
