# Supplementary material for: Toxicity Effects of Polystyrene Nanoplastics with Different Sizes on Freshwater Microalgae Chlorella vulgaris
Source: Molecules. 2023 May 8;28(9):3958. doi: 10.3390/molecules28093958 (PMC10180472; doi:10.3390/molecules28093958)
Supplement: Supplementary file 1 [file molecules-28-03958-s001.zip › molecules-2382556-supplementary.pdf]

Supplementary information

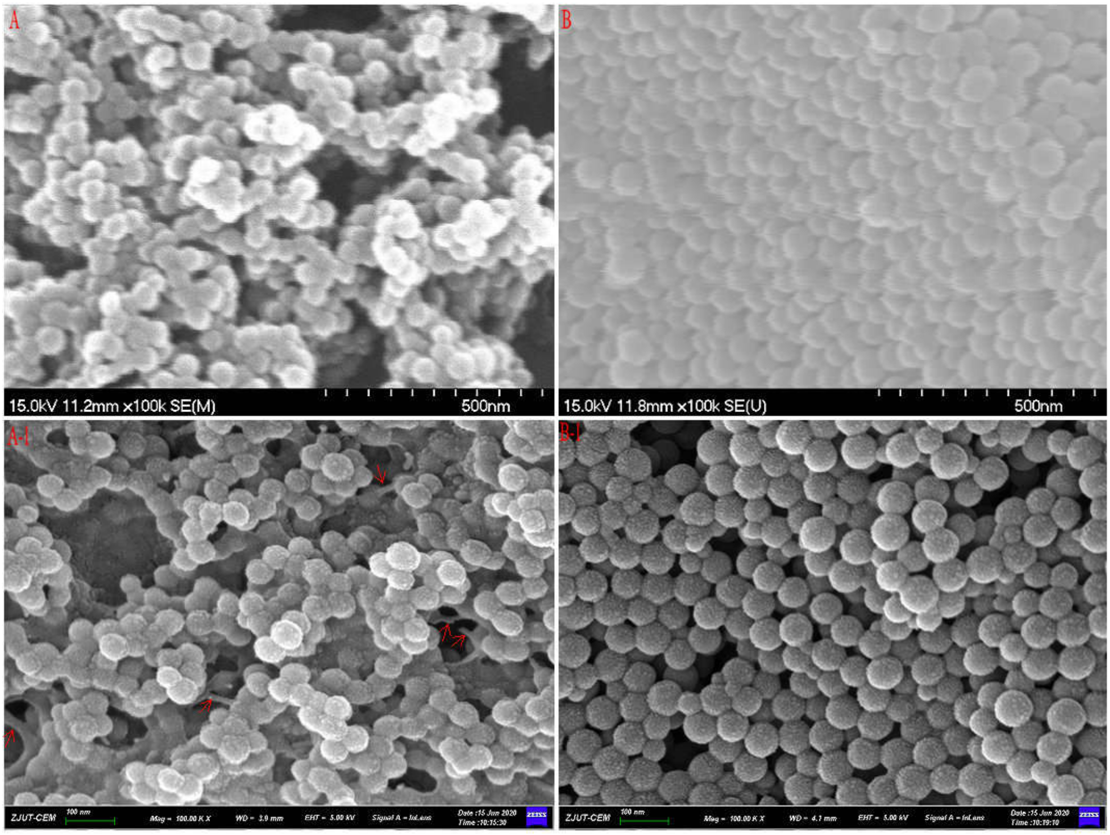

Figure S1. The SEM images of 50 nm PS-NPs (left) and 70 nm PS-NPs (right) (above: raw materials; below: PS-NPs after 72 h exposure in BG-11, red arrows represent the deformation of PS-NPs).

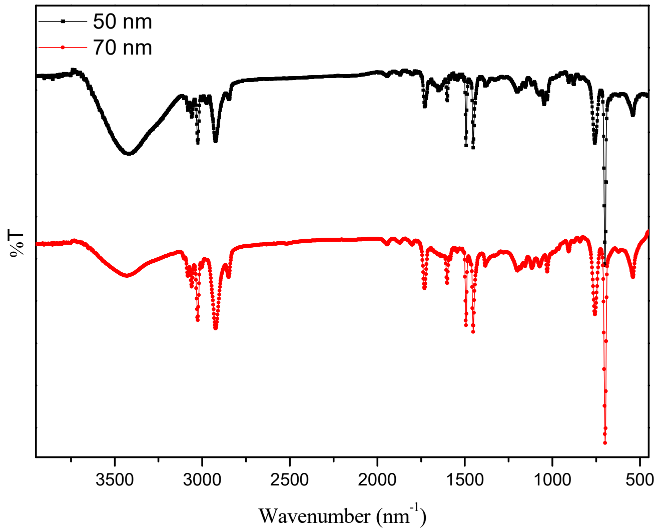

Figure S2. FTIR characterization of primary 50 nm and 70 nm PS-NPs

Table S1. The EC_{50} values for algae growth and chlorophyll a after exposing to 50 and 70 nm PS-NPs (0-50 mg/L) for 72 h.

PS-NPs size	Growth EC_{50}(72h)	Growth 95% confidence interval Lower limit	Growth 95% confidence interval Upper limit	Chl-a EC_{50}(72h)	Chl-a 95% confidence interval Lower limit	Chl-a 95% confidence interval Upper limit
50 nm	19.89	15.58	27.44	17.52	13.31	24.72
70 nm	>50	-	-	>50	-	-

EC_{50} was calculated according to their inhibition rates in respect to the control.

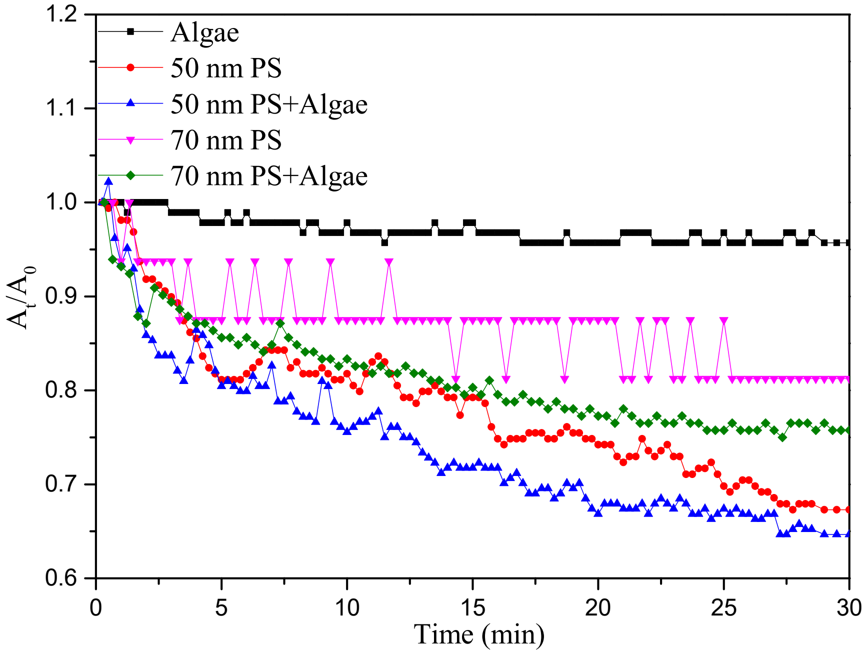

Figure S3. Effect of PS-NPs on sedimentation for algal cell（20 mg/L PS-NPs）
